# Total synthesis of grayanane natural products

**DOI:** 10.3762/bjoc.18.181

**Published:** 2022-12-12

**Authors:** Nicolas Fay, Rémi Blieck, Cyrille Kouklovsky, Aurélien de la Torre

**Affiliations:** 1 Institut de Chimie Moléculaire et des Matériaux d’Orsay (ICMMO), Université Paris-Saclay, CNRS, 15, rue Georges Clémenceau, 91405 Orsay Cedex, France

**Keywords:** enantioselectivity, grayananes, oxidation, 1,2-shift, total synthesis

## Abstract

Grayananes are a broad family of diterpenoids found in *Ericaceae* plants, comprising more than 160 natural products. Most of them exhibit interesting biological activities, often representative of *Ericaceae* use in traditional medicine. Over the last 50 years, various strategies were described for the total synthesis of these diterpenoids. In this review, we survey the literature for synthetic approaches to access grayanane natural products. We will focus mainly on completed total syntheses, but will also mention unfinished synthetic efforts. This work aims at providing a critical perspective on grayanane synthesis, highlighting the advantages and downsides of each strategy, as well as the challenges remaining to be tackled.

## Introduction

The *Ericaceae* are a large plant family, with over 4250 known species all around the world [1]. While *Ericaceae*’s toxicity has been known since at least 400 BC, barks, leaves and flowers from these plants are still commonly used in traditional medicine in Asia, Europe and America, mainly for their anti-inflammatory and analgesic properties, but also to treat different conditions (e.g. arthritis, hypertension, diabetes, lung, liver and gastrointestinal disorders), and for crop protection [2].

Among natural products formed by *Ericaceae*, grayananes are a wide diterpenoid family whose biological activities are often representative of *Ericaceae*’s use in traditional medicine [3–5]. In particular, many grayananes were found to have analgesic [6], antifeedant [7–9], antituberculosis [10], cytotoxic [11] and antioxidant [12] properties. The grayanane family comprises more than 160 natural products, and new members are isolated every year. For instance, 13 new grayanane natural products were reported since 2020 [13–16].

**Figure 1:**
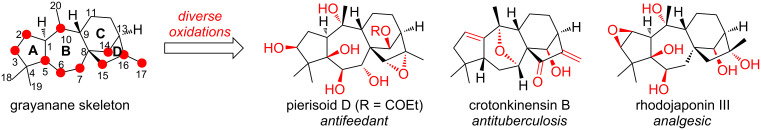
General structure of grayanane natural products.

Grayanane diterpenoids all share the same tetracyclic skeleton, with 5, 7, 6 and 5-membered carbocycles commonly named A, B, C and D (Figure 1). The diversity in this family arises from different oxidation states at positions 2, 3, 5, 6, 7, 10, 14, 15, 16, and 17 which can bear free, acylated or glycosylated alcohol, olefin, ketone or epoxide functionalities.

**Scheme 1:**
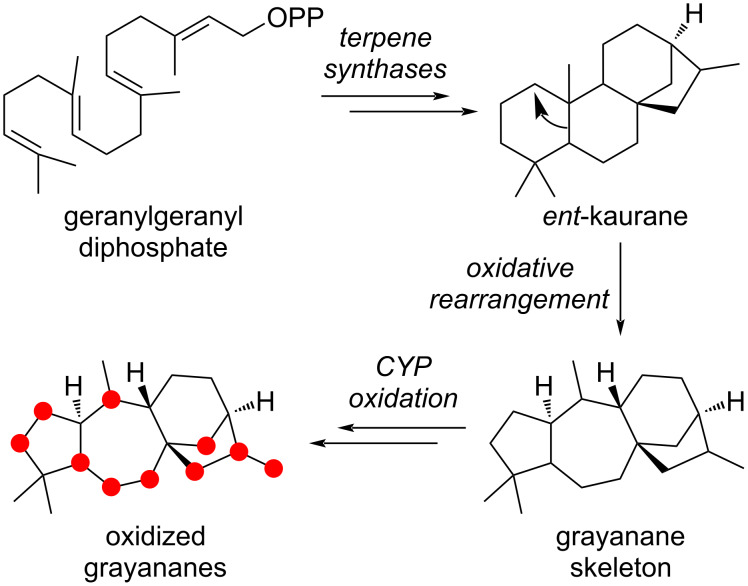
Grayanane biosynthesis.

From a biosynthetic point of view, grayananes arise from an oxidative rearrangement of the *ent*-kaurane skeleton (Scheme 1). The diversity is generated by cytochromes P450 (CYP) enzymatic oxidation of the grayanane skeleton [17].

The biological activities and low extraction yields have prompted various research groups to undertake the total synthesis of grayanane natural products. In this work, we will survey the literature for synthetic routes to grayanane diterpenoids, including uncompleted approaches. The review is chronologically organized, starting from the earliest synthetic efforts from 1971 to the latest in 2022. In a last part we will present unfinished syntheses.

## Review

### Early syntheses by Matsumoto and Shirahama

**Scheme 2:**
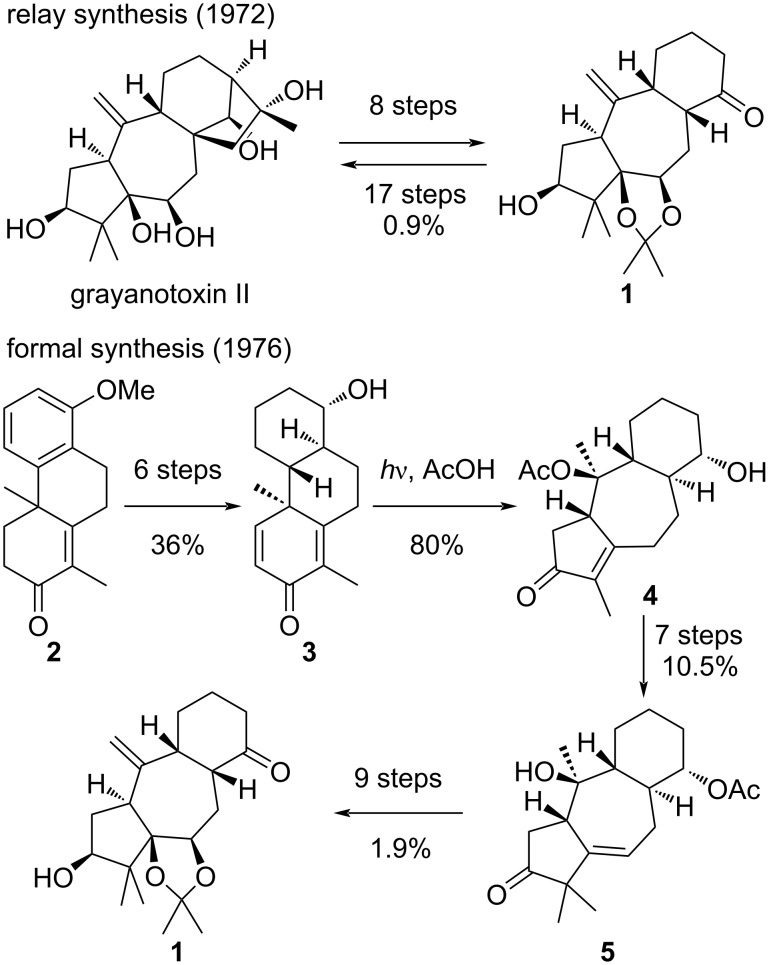
Matsumoto’s relay approach.

The first synthetic approach towards a grayanane natural product was reported by Matsumoto in the 70s, using a relay approach. The authors first reported in 1972 the synthesis of grayanotoxin II from a degradation product **1** obtained in a few steps from grayanotoxin II itself (Scheme 2) [18]. Later on in 1976 [19], the same authors reported the synthesis of intermediate **1** from the known phenanthrene derivative **2** [20]. The tricyclic compound **2** was converted to **3** through a 6-step sequence involving reduction of the aromatic ring and oxidation of the enone to a dienone. The resulting dienone **3** underwent a key photoinduced santonin-like rearrangement in the presence of acetic acid, furnishing **4** in high yield. It should be noted that the group of Hiraoka had previously reported a similar rearrangement for the synthesis of a grayanane-type skeleton [21]. Further methylation and protecting group interconversions lead to an advanced tricyclic structure **5**, which could be further elaborated into relay intermediate **1**.

Although Matsumoto’s approach does not fit with the requisites of modern organic synthesis, as it requires over 40 steps to synthesize grayanotoxin II and was performed in racemic form (in the case of the formal synthesis), it represents an impressive piece of synthetic work. Achieving the synthesis of such a complex structure represented a challenging puzzle at that time, and was brilliantly solved by Matsumoto and co-workers.

Almost 20 years later, in 1994, Shirahama’s group published the total synthesis of another member of the grayananes: grayanotoxin III [22]. One of the keys of their synthesis was the stereocontrolled 7-membered ring closure through a SmI_2_-promoted pinacol coupling. This reaction had been previously described by the same group and proved to be highly effective [23]. For rings A, C and D, other SmI_2_-promoted steps were employed, giving respectively the cyclopentane A ring moiety and the bicyclo[3.2.1]octane CD ring system with the correct configuration.

**Scheme 3:**
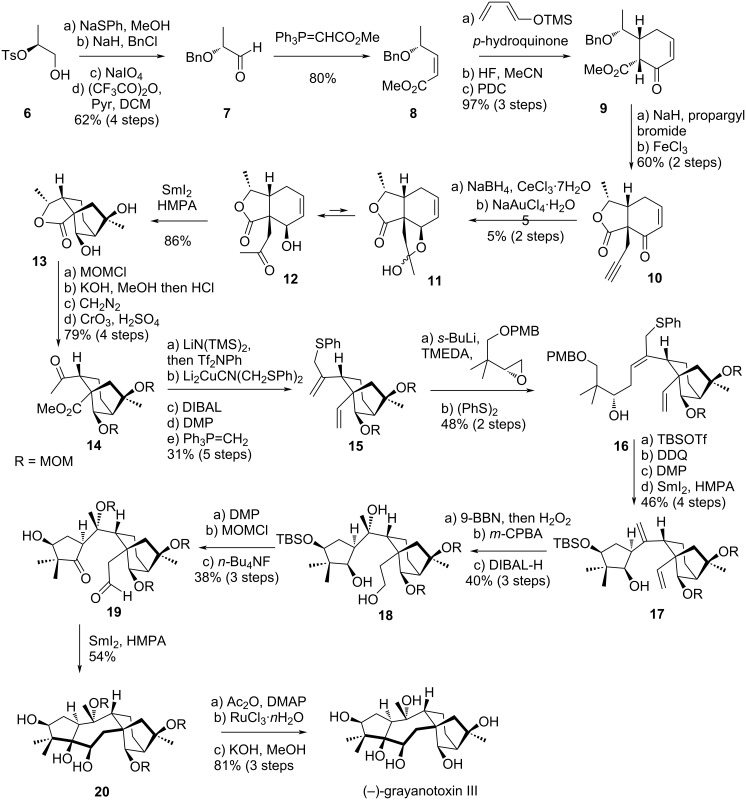
Shirahama’s total synthesis of (–)-grayanotoxin III.

The synthesis started from commercially available (*S*)-2-((*p*-toluenesulfonyl)oxy)-1-propanol (**6**) which was converted to (*R*)-2-(benzyloxy)propionaldehyde (**7**) by a sequence involving formation of the a phenyl sulfide through an epoxide intermediate, protection of the secondary alcohol as a benzyl ether, oxidation of the sulfur and Pummerer rearrangement (Scheme 3). A Wittig reaction gave compounds **8**, as a 10:1 separable diastereomeric mixture. A diastereoselective Diels–Alder cycloaddition followed by oxidation of the resulting epimeric mixture gave substituted cyclohexanone **9**, corresponding to the future C ring [24]. After deprotonation, the C^3^ position was stereoselectively alkylated using propargyl bromide, and the benzyl protecting group was cleaved with FeCl_3_, leading to spontaneous lactone closure. A Luche reduction stereoselectively converted enone **10** to the corresponding allylic alcohol, followed by a Au-catalyzed alkyne hydration, providing hemiketal **11**. This intermediate was in equilibrium with hydroxy-ketone **12**, which was suitable for a SmI_2_-promoted cyclization, affording intermediate **13** selectively, already bearing rings C and D. The selectivity was achieved by chelation of the Sm(III) intermediate with hydroxy groups present on the structure. As the direct coupling with the A-ring precursor failed, a strategy to build this part was developed, starting with a sequence involving a protection of the alcohols as MOM ethers, lactone hydrolysis, esterification, and Jones oxidation, affording intermediate **14** with a good 79% yield over 4 steps. Next, the methyl ketone was converted to an enol triflate, and then coupled with Li_2_CuCN(CH_2_SPh)_2_. A reduction of the ester with DIBAL, followed by Dess–Martin oxidation and Wittig reaction lead to the formation of **15**. This intermediate was coupled with an (*R*)-epoxide in presence of *s-*BuLi, and intermediate **16** with *E* configuration was then obtained by a (PhS)_2_-accelerated 1,3-sulfide shift. The A ring was then cyclized by a sequence consisting of protection of the alcohol, oxidative cleavage of the PMB protecting group, Dess–Martin oxidation, and SmI_2_-induced cyclization. This last step was highly selective, giving solely the intermediate **17**. The synthesis was then pursued by the hydroboration–oxidation of the monosubstituted alkene, followed by stereoselective epoxidation of the 1,1-disubstituted olefin and reductive epoxide ring-opening giving triol **18**. After oxidation of the primary and the secondary alcohols with Dess–Martin periodinane, the remaining tertiary alcohol was protected as a MOM ether and the silyl ether protecting group was removed. The obtained intermediate **19** was then a suitable starting material for the SmI_2_-promoted pinacol coupling, directed by the free hydroxy group, affording a complete selectivity in the formation of the 7-membered ring B. The synthesis of grayanotoxin III was then achieved by acetylation of the secondary alcohols, oxidative cleavage of the MOM protecting groups followed by hydrolysis of the acetyl protecting groups, affording the desired product with spectral data identical to reported natural samples.

Shirahama’s synthesis was an illustrative example that rings A, B and the bicycle CD of grayananes could all be obtained by SmI_2_-promoted steps, obtaining excellent selectivity in all cases. Alternatively, the same year, the authors published a vinyl radical cyclization occurring in presence of *n-*Bu_3_SnH, providing a stereoselective access to the bicyclo[3.2.1]octane unit corresponding to the CD rings [25].

### Newhouse’s synthesis of principinol D

In 2019, Newhouse’s group published a total synthesis of principinol D [26], a compound isolated from *Rhododendron principis* in 2014 [27–28]. Compared to grayanotoxin III, principinol D displays an inverse configuration at C^1^ as well as an *exo*-olefin at C^10^–C^20^. The strategy developed by the group relied on the obtainment of two distinct fragments, a racemic bicyclo[3.2.1]octane unit **25** corresponding to rings C and D, and an enantioenriched cyclopentyl aldehyde derivative **29**, corresponding to ring A. These fragments were successfully coupled under basic conditions, and the ring B was later reductively closed using SmI_2_.

**Scheme 4:**
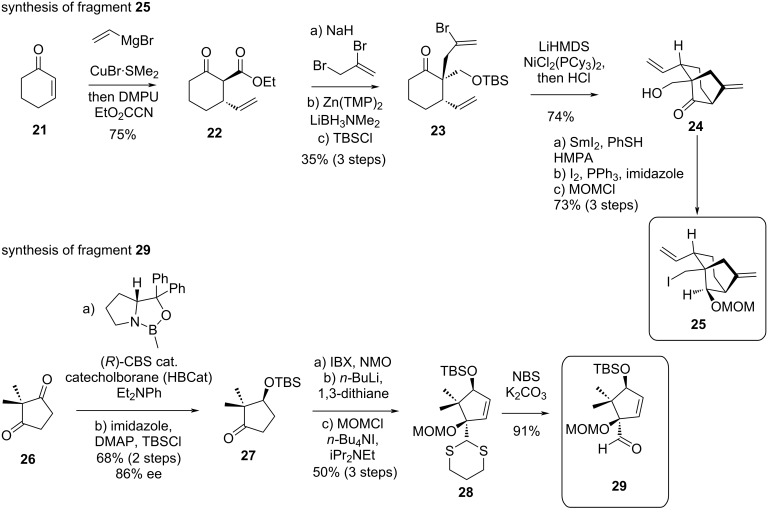
Newhouse’s syntheses of fragments **25** and **29**.

The synthesis of fragment **25** began with commercially available cyclohexenone (**21**), which underwent a copper-catalyzed vicinal difunctionalization with vinylmagnesium bromide and DMPU and trapping using methyl cyanoformate, leading to the formation of ketoester **22** (Scheme 4). This intermediate was then allylated, the ester group selectively reduced with Zn(TMP)_2_ and LiBH_3_NMe_2_ and the resulting primary alcohol was protected as a TBS ether, providing intermediate **23** as a single diastereomer. This key intermediate **23** was then submitted to a Ni-catalyzed α-vinylation and direct TBS deprotection giving the bicyclo[3.2.1]octane subunit with a good yield of 74%. A sequence involving diastereoselective reduction of ketone **24** with SmI_2_, Appel reaction to convert the primary alcohol to the corresponding primary alkyl iodide followed by a MOM protection of the secondary alcohol afforded fragment **25** with 73% yield over 3 steps. On the other hand, the enantioenriched cyclopentyl aldehyde fragment **29** was obtained starting from commercially available 2,2-dimethylcyclopentane-1,3-dione (**26**). The dione was submitted to a sequence involving a monoreduction, protection of the alcohol as a TBS ether, α,β-desaturation, dithiane addition, MOM protection and dithiane deprotection.

**Scheme 5:**
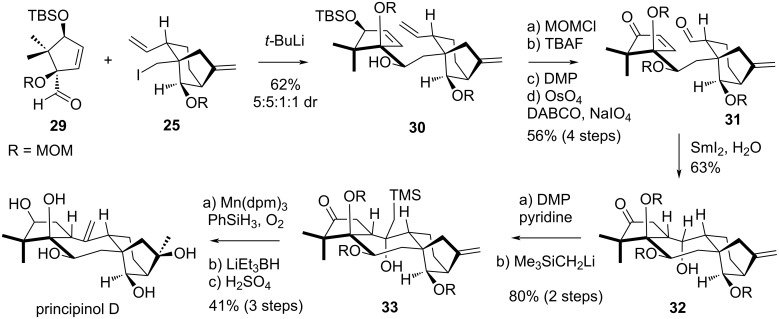
Newhouse’s total synthesis of principinol D.

The fragment **25** was lithiated with *t-*BuLi and the fragment **29** was then added, forming the coupling product as a 5:5:1:1 separable mixture of diastereomers (Scheme 5). The desired diastereomer **30** was isolated with a yield of 26%. The secondary alcohol was protected as a MOM ether and the allylic silyl ether was converted to an enone. A selective oxidative cleavage, only affecting the monosubstituted alkene, led to the formation of **31**, which underwent a key SmI_2_-promoted seven-membered ring closure, giving a single diastereomer. The stereochemistry and absolute configuration of the obtained tetracyclic structure **32** was confirmed by NOESY NMR and X-ray crystallography. Some additional modifications were required on the structure to synthesize principinol D: oxidation of the secondary alcohol to the corresponding ketone was achieved using Dess–Martin periodinane with a pyridine buffer. Addition of Me_3_SiCH_2_Li efficiently afforded the Peterson adduct **33**. The 1,1-disubtituted alkene was then submitted to Mukaiyma hydration to form the tertiary alcohol, in presence of Mn(dpm)_3_, PhSiH_3_ and O_2_. Then, the ketone was selectively reduced in the presence of LiEt_3_BH, while the Peterson adduct was eliminated concurrently, upon heating. Finally, treatment with H_2_SO_4_ allowed total deprotection of the MOM ethers, leading to the formation of principinol D, in complete correspondence with reported spectral data.

Newhouse’s synthesis represents an efficient access to grayananes, relying on two accessible fragments. Remarkably, although the strategy is different from that of Shirahama as it involves different retrosynthetic disconnections, it makes use of similar tactics through the use of a SmI_2_-promoted cyclization. This first total synthesis of principinol D, in 19 steps as the longest linear sequence, is asymmetric even though a separation of the mixture of diastereomers resulting from fragment coupling is necessary. The SmI_2_-mediated reductive ring-closure of the 7-membered ring is among the most remarkable steps of the synthesis, along with the Ni-catalyzed formation of the bicyclo[3.2.1]octane unit. It should be noted that due to the SmI_2_-mediated ring-closure’s stereochemical outcome, this synthesis can only be applied to compounds with an *R*-configured C^1^ stereocenter, which are epimers of the vast majority of grayanane structures.

### Ding’s synthesis of rhodomolleins XX and XXII

Shortly after Newhouse’s synthesis, Ding’s group reported a synthetic strategy to access rhodomollein XX and XXII [29–30]. These natural products have the particularity of displaying an enone moiety on ring A. Ding’s approach involves the construction of a tetracyclic structure where rings A and B had the correct arrangement, while rings C and D form a bicyclo[2.2.2]octane structure [31]. The correct bicyclo[3.2.1]octane structure was obtained after a key reductive epoxide opening/Dowd–Beckwith rearrangement cascade.

**Scheme 6:**
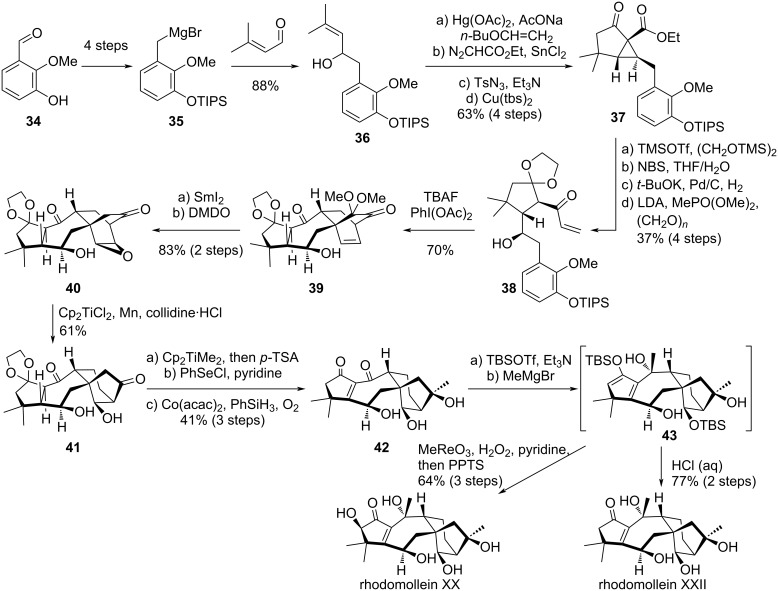
Ding’s total synthesis of rhodomolleins XX and XXII.

The synthesis started from 3-hydroxy-2-methoxybenzaldehyde (**34**), which was converted into Grignard reagent **35** and added onto 3-methylbut-2-enal (Scheme 6). A sequence involving Claisen rearrangement, Roskamp homologation, diazo transfer and intramolecular cyclopropanation led to intermediate **37**. The hydroxy group on C^6^ was introduced after cyclopropane ring-opening, ketone protection, epoxidation and reductive ring-opening of the resulting epoxide. A one-pot β-keto phosphonate formation/Horner–Wadsworth–Emmons reaction with formaldehyde afforded **38**, a precursor for the key oxidative dearomatization-induced Diels–Alder cycloaddition. Treatment of **38** with TBAF followed by PhI(OAc)_2_ led to the formation of **39**, having the A and B ring correctly arranged. The product was obtained in 70% yield, along with 25% of an undesired diastereoisomer. The dimethoxy functionality was reduced in the presence of Kagan’s reagent and DMDO could induce an epoxidation on the strained olefin. From intermediate **40**, the key reductive epoxide opening/Dowd–Beckwith rearrangement cascade could be performed in the presence of an in situ-generated Ti(III) catalyst. The main side-product of this reaction was due to a simple reductive opening of the epoxide (15%). From **41** having the correct tetracyclic skeleton, a transient protection followed by Petasis olefination, deprotection, selenide-mediated α,β-dehydrogenation and Mukaiyama oxidation afforded an advanced intermediate **42** bearing most of the target’s functionalities. A sequence of enol-ether formation/Grignard addition lead to intermediate **43**, from which simple acidic treatment led to rhodomollein XXII, while α-oxidation in the presence of rhenium oxide followed by acidic work-up afforded rhodomollein XX.

Interestingly, Ding’s synthesis constitutes an efficient approach (22 and 23 steps) to access grayananes with a cyclopentenone moiety on the A ring. It should be noted that although this is a racemic synthesis, intermediate **37** was also synthesized in enantioenriched form using a chiral copper catalyst for the cyclopropanation and a chiral auxiliary on the ester moiety. Moreover, the same group reported a related approach for the synthesis of various diterpenoids including rhodomollanol, an *abeo*-grayanane natural product [32–33].

### Luo’s synthesis of grayanotoxin III, principinol E and rhodomollein XX

**Scheme 7:**
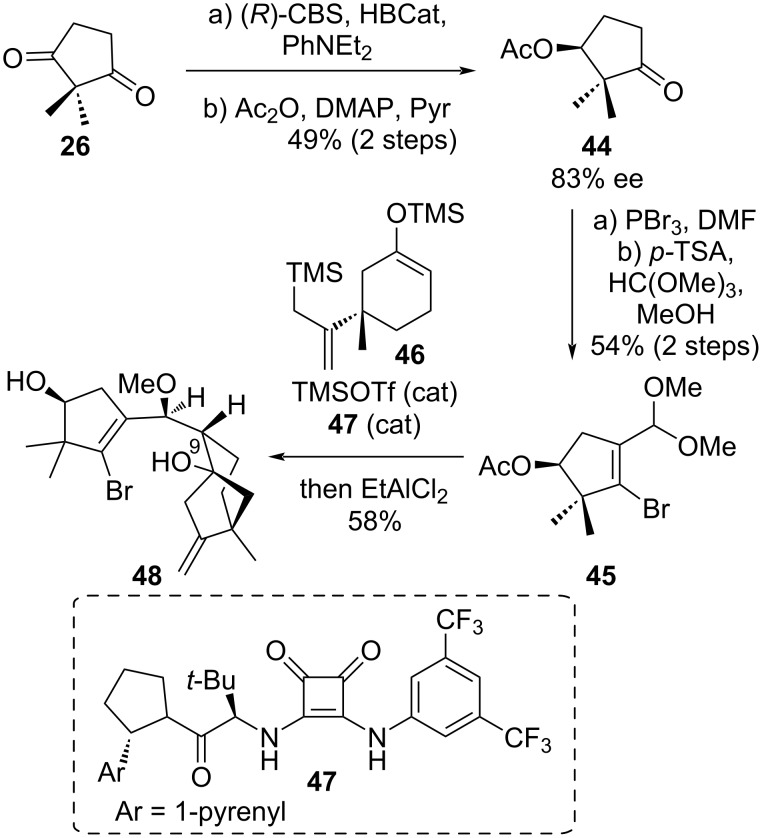
First key step of Luo’s strategy.

In 2022, Luo et al. described an efficient and enantioselective synthetic route based on a convergent strategy to accomplish the synthesis of principinol E, grayanotoxin III and rhodomollein XX [34]. The key steps include i) a tandem reaction combining organocatalytic Mukaiyama aldol and intramolecular Hosomi–Sakurai reactions in a one-pot manner; ii) a 7-membered cyclization based on a bridgehead tertiary carbocation intermediate forging the B ring; iii) redox manipulations and a 1,2-migration as final steps. The synthesis started from (*S*)-ketone **44** which was prepared via asymmetric CBS reduction of diketone **26** (Scheme 7). Firstly, this (*S*)-ketone **44** was transformed into dimethylacetal **45** by Vilsmeier reaction followed by aldehyde protection in 54% yield over two steps. A Mukaiyama aldol reaction between trimethylsilyl enol ether **46** and dimethylacetal **45** followed by Sakurai cyclization provided an inseparable mixture of C^9^ epimers (dr = 2:1). A catalyst optimization showed that chiral squaramide **47** developed by Jacobsen’s group significantly accelerated the Mukaiyama reaction compared to TMSOTf or TiCl_4_ thanks to chiral hydrogen bond-donor effect [35]. After Sakurai cyclization promoted by EtAlCl_2_, the desired product **48** was obtained with the required diastereoselectivity in 58% on a 3 g scale.

**Scheme 8:**
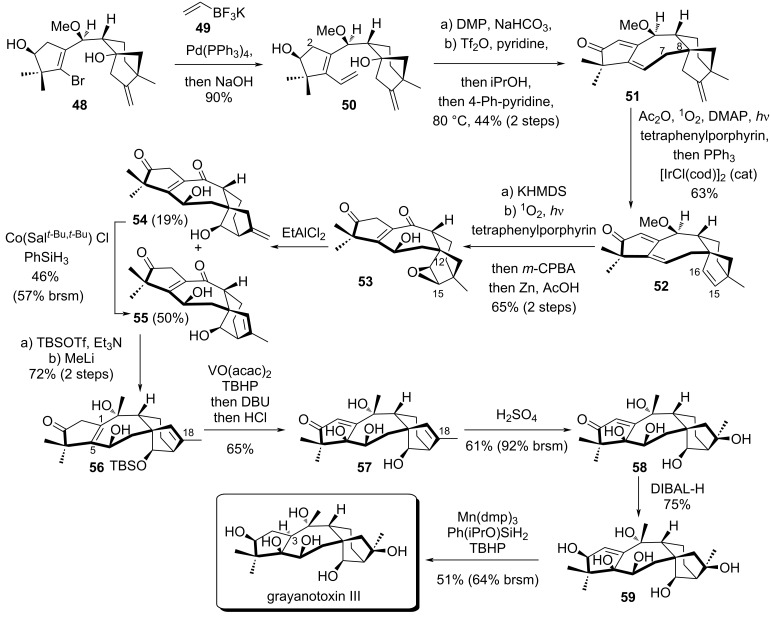
Luo’s total synthesis of grayanotoxin III.

Subsequently, vinyl halide **48** was converted to diene **50** by Suzuki coupling with potassium vinyltrifluoroborate (**49**) in 90% yield (Scheme 8). The C^7^–C^8^ bond formation from a bridgehead carbocation was a real challenge to close the 7-membered ring. To achieve this, the secondary alcohol was oxidized by DMP, the tertiary alcohol was triflated, 4-phenylpyridine was added and the mixture was heated at 80 °C for 14 h. The intermediate carbocation was trapped by the terminal olefin, generating a dienone **51** after deprotonation at the relatively acidic position C^2^. A singlet oxygen ene reaction involving the electron-rich olefin allowed the formation of an aldehyde, which was directly cleaved by an iridium-catalyzed deformylation, affording **52** in one-pot [36]. Deprotonation with KHMDS allowed the formation of an electron-rich diene which could again react with singlet oxygen by diastereoselective cycloaddition followed by C^15^–C^16^ epoxidation with *m-*CPBA. Reductive cleavage of the O–O bond by Zn/AcOH treatment afforded epoxide **53** as a single diastereomer in two steps and 65% yield. The correct bicyclo[3.2.1]octane was obtained by Wagner–Meerwein epoxide rearrangement promoted by EtAlCl_2_. Two separable alkene regioisomers **54** and **55** were obtained in 19% and 50% yield, respectively. A metal-catalyzed hydrogen atom transfer (MHAT) allowed **54** to be partially converted to **55** via Shenvi’s isomerization [37]. Selective TBS protection on the bicyclo[3.2.1]octane moiety and ketone methylation gave access to **56**. Directed C^1^–C^5^ vanadium-mediated epoxidation followed by DBU treatment and TBS deprotection afforded **57** in one pot. The tertiary alcohol **58** was obtained as a single diastereomer after hydration of position C^18^. Subsequent reduction with DIBAL-H gave the desired alcohol on the A ring in 75% yield. The C^3^ epimer was also obtained in 4% yield and confirmed by X-ray diffraction. Hydrogenation of the sterically hindered C^1^–C^2^ alkene was accomplished using a combination of Mn(dpm)_3_ and Ph(iPrO)SiH_2_, providing grayanotoxin III in 51% yield.

**Scheme 9:**
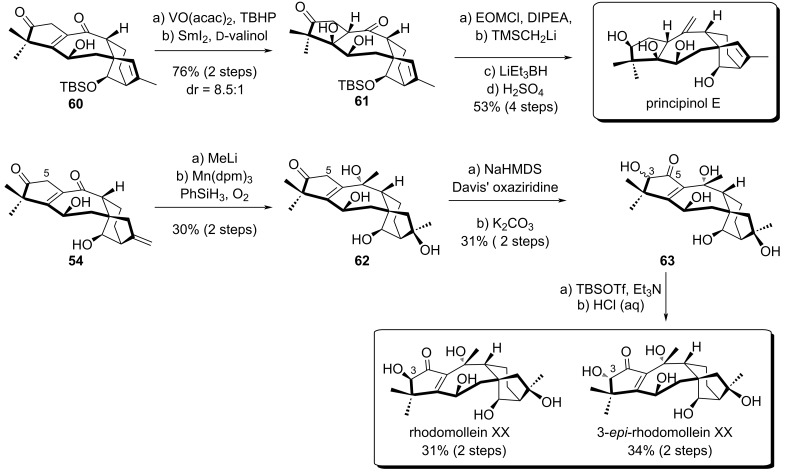
Synthesis of principinol E and rhodomollein XX.

The authors also achieved the synthesis of principinol E and rhodomoline XX by slight modifications of the late-stage functional group transformations. The synthesis of principinol E was performed in 6 steps starting from **60**, which was obtained by protection of **55** (Scheme 9). As before, directed epoxidation of C^1^–C^5^ in compound **60** was employed to form an α,β-epoxyketone intermediate which underwent SmI_2_ reduction at −78 °C, giving access to the desired *syn* diastereomer with good selectivity (dr = 8.5:1). The free alcohol was then protected with an ethoxymethyl ether (EOM). Finally, Peterson olefination, stereoselective carbonyl reduction and TBS deprotection afforded principinol E in 53% yield over 4 steps. For the synthesis of rhodomollein XX, addition of methyllithium to **54** followed by Mukaiyama hydration using Mn(dpm)_3_ afforded **62**. To access rhodomollein XX, an additional oxidation state at the C^2^ position was required. Luo's team used Davis’ oxaziridine followed by treatment with K_2_CO_3_ to equilibrate the hydroxyketone, delivering an inseparable epimeric mixture (1:3) of **63** in 31% overall yield. An additional TBS protection allowed separation of the epimers. After acidic treatment, pure rhodomollein XX and 3-*epi*-rhodomollein were obtained in 31% and 34% yield in two steps, respectively.

Luo’s synthetic work offers a powerful approach to synthesize grayanane natural products. The synthesis is both efficient (18 to 20 steps) and flexible, as demonstrated by the synthesis of 3 natural products, using pivotal intermediates **54** and **60**.

### Other synthetic efforts

**Scheme 10:**
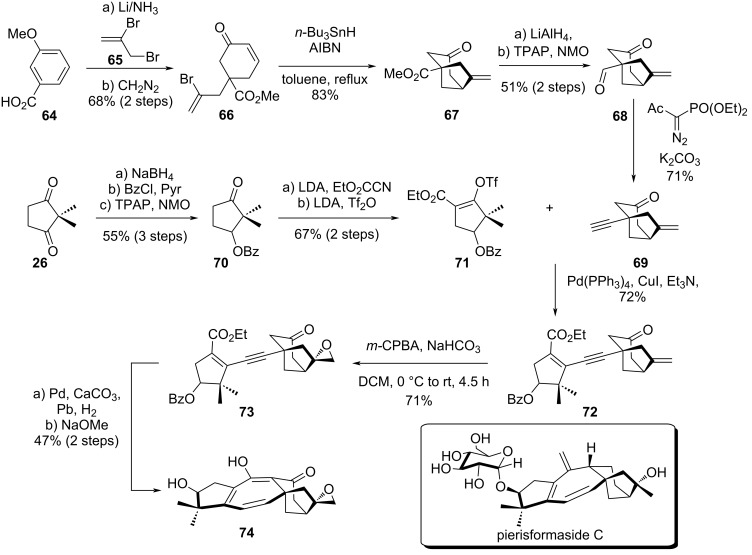
William’s synthetic effort towards pierisformaside C.

In 2011, Williams and co-workers reported an efficient synthetic route detailing the central core construction of pierisformaside C, which is the first grayanane isolated displaying three central double bonds (Scheme 10) [38]. The team’s objective was to develop a synthetic pathway also giving access to several diterpene glycosides close to pierisformaside C in order to study the biological activity of this family. Their strategy was based on a common forward intermediate and a late construction of the central seven-membered ring.

In the beginning of the synthesis, the authors followed the strategy developed previously by Marinovi’s group to form the bicyclo[3.2.1]octane moiety [39]. The synthesis started from **64** with a one-pot Birch reduction/alkylation with vinyl bromide **65**, affording **66** in 68% yield over two steps. Next the construction of the bicylo[3.2.1]octane **67** was achieved by a radical cyclization using *n*-Bu_3_SnH in refluxing toluene. A sequence involving an ester reduction, Ley–Griffith oxidation and Seyferth–Gilbert homologation with Bestmann–Ohira reagent allowed to obtain the alkynyl bicylo[3.2.1]octane **69**. On the other hand, the five-membered triflate **71** was synthesized from diketone **26** in 5 steps and 37% overall yield. Both fragments were assembled by a Sonogashira cross-coupling, affording **72** in 72% yield. In a first attempt, TBS protection was considered on the bicylo[3.2.1]octane. However, later in the strategy, the deprotection presented some difficulties, and the authors decided to investigate the use of a free ketone. The partial hydrogenation of alkyne **72** proved to be inefficient, due to a lack of chemoselectivity involving competitive olefin reduction on the bicylo[3.2.1]octane. To overcome the over-oxidation, **72** was treated with *m*-CPBA, providing epoxide **73** as the main product in 71% yield (dr = 6:1). Lindlar hydrogenation of the alkyne and cyclization proceeded smoothly, and the tetracyclic skeleton **74** was obtained in moderate yield. However, the synthesis of pierisformaside C was never completed. The missing transformations include the removal of the ketone on the C ring, epoxide reductive opening, formation of the B ring *exo*-olefin, and glycosylation.

In 2021, Hong et al. presented a synthetic effort focused on the synthesis of rhodojaponin III B–C rings [40]. The authors employed a Mn(III)-mediated intramolecular radical cyclization of an alkynyl ketone as the key step.

**Scheme 11:**
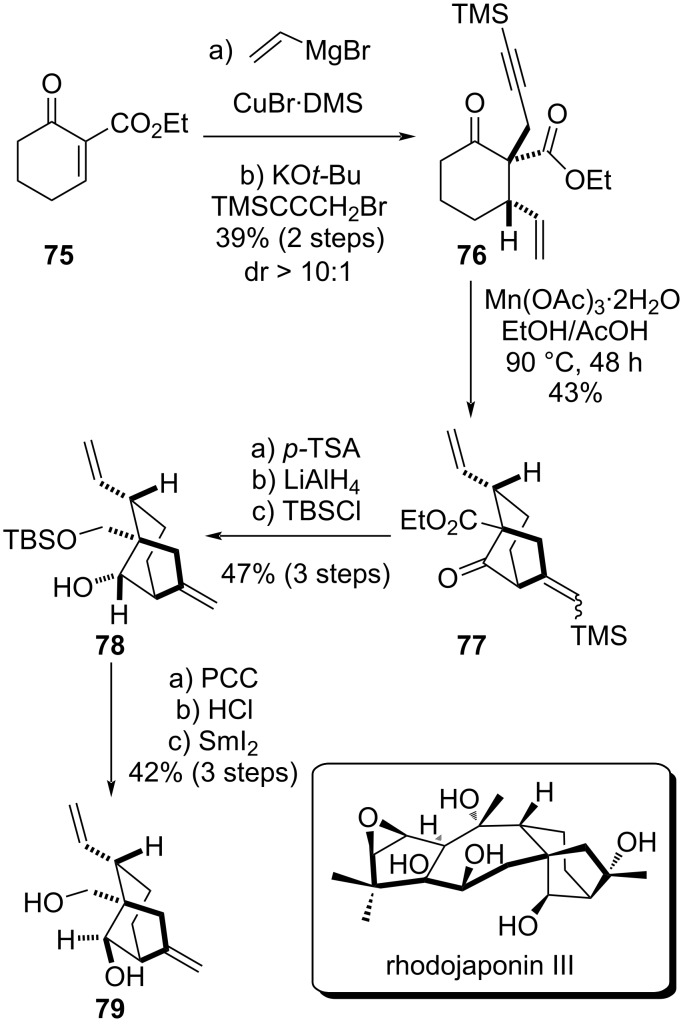
Hong’s synthetic effort towards rhodojaponin III.

The synthesis started by a Cu-catalyzed conjugate addition of the vinyl Grignard reagent, followed by TMS α-propargylation under basic conditions, affording the TMS-alkynyl ketone **76** as the major diastereomer (Scheme 11). Originally a Au-catalyzed Conia-ene-type cyclization, classically considered as a reliable method for the construction of bridged bicyclic structures [41], was envisaged. However, using a gold(I) catalyst the desired 5-*exo*-*dig* cyclization failed and only a 6-*endo*-*dig* cyclization was observed. Thus, Hong et al. explored a Mn(III)-mediated radical cyclization, an approach which had been previously reported by Jia and co-workers during the synthesis of glaucocalyxin A [42]. After treatment with Mn(OAc)_3_, the desired 5-*exo*-*dig* cyclization product **77** was obtained in 43% yield as an *E*/*Z* mixture. The TMS group was removed under acidic conditions. Then, a wide range of reducing agents was explored for the stereoselective ketone reduction. However, only the undesired diastereomer was obtained. Knowing that the correct diastereomer could be obtained in the presence of a primary alcohol instead of the ester moiety, as described by Newhouse previously [26], the authors performed a complete reduction of the carbonyl moieties using LiAlH_4_ and TBS protection of the primary alcohol, affording intermediate **78** with the wrong configuration at the secondary alcohol stereocenter. After reoxidation and deprotection of the primary alcohol, SmI_2_ reduction finally afforded the desired diastereomer **79**. This synthetic work highlights again the challenge of the bicyclo[3.2.1]octane construction, especially regarding the diastereocontrol at the secondary alcohol moiety. To date, this approach towards rhodojaponin III was never concluded.

## Conclusion

Over the past 50 years, the synthesis of grayanane natural products has attracted the interest of many synthetic chemists, leading to the development of five total synthesis approaches, as well as two unfinished syntheses [43]. Table 1 summarizes the completed total syntheses in terms of steps, yields, advantages and drawbacks. The clear superiority of recent syntheses beautifully showcases the progresses in the field of organic synthesis over the last decades. Among the last three syntheses, Newhouse’s work has the huge advantage of being convergent, allowing a short synthesis (19 steps longest linear sequence). On the other hand, this strategy is limited to the synthesis of C^1^ epimers of the grayanane general structure. Although linear and thus requiring a few more steps, Ding’s syntheses still provide higher yields than Luo’s and Newhouse’s. Still, this strategy is also limited in terms of accessible structures, as only C^1^–C^5^ dehydrogenated natural products are obtained. It should be noted that despite this, a similar approach was applied by Ding’s group to the synthesis of various diterpenoids. Finally, Luo’s synthesis has the advantage of being more flexible, as it allows the synthesis of the general structure of grayananes. Nevertheless, this approach is somehow lower yielding than the previous ones. Regarding enantioselectivity, both Newhouse’s and Luo’s syntheses rely on an enantioenriched precursor obtained in 86% ee. On the other hand, while Ding’s syntheses were performed in racemic form, the authors showed that a key intermediate could be obtained enantioselectively (93% ee) by a combination of a chiral catalyst and chiral auxiliary, although requiring extra steps for auxiliary installation and cleavage.

**Table 1:** Summary of previous syntheses.

Synthesis	Number of steps	Overall yield	Advantages	Drawbacks

Matsumoto (1972–1976) [18–19]	>40	<0.0005%	–	many steps, racemic
Shirahama (1994) [22]	38	0.05%	enantioselective	many steps
Newhouse (2019) [26]	19	0.4%	convergent, few steps	limited to C^1^ epimers of the general structure
Ding (2019) [31]	22–23	1.2–1.4%	few steps, highest yielding	limited to C^1^–C^5^ dehydrogenated grayananes
Luo (2022) [34]	18–20	0.01–0.5%	few steps, flexible	lower yields

**Scheme 12:**
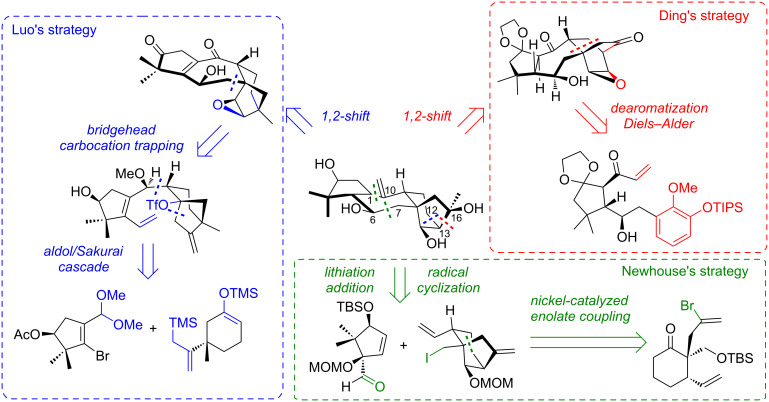
Recent strategies for grayanane synthesis.

Scheme 12 summarizes the last 3 synthetic strategies for grayanane synthesis. Each group proposes different disconnections. Newhouse identified a C^1^–C^10^ disconnection for a radical cyclization and a C^6^–C^7^ for an organolithium addition, allowing a convergent approach with A ring and C–D rings as key fragments. In this case, the bicyclo[3.2.1]octane is obtained by a Ni-catalyzed enolate/alkenyl bromide coupling. While both Ding and Luo propose a 1,2-shift relying on epoxide ring-opening as key step, each group proposes a different disconnection. For Ding, the rearrangement forms bond C^13^–C^16^ through a Ti(III)-mediated reductive epoxide opening/Dowd–Beckwith rearrangement cascade. This allows to have a bicyclo[2.2.2]octane precursor, which can be obtained by a dearomatization/Diels–Alder cascade. For Luo, the 1,2-shift forms bond C^12^–C^13^ through a cationic Wagner–Meerwein-type rearrangement. The B ring is obtained by a key bridgehead carbocation trapping, while the skeleton arises from an A ring fragment and a C ring fragment, assembled by a diastereoselective aldol/Sakurai cascade.

In recent years, important advances have been made regarding the synthesis of grayanane natural products, paving the way for deeper biological activity evaluation. Nevertheless, important challenges still need to be tackled. A highly enantioselective synthesis is still desirable, as the only synthesis offering >90% ee relies on the combination of chiral ligands and chiral auxiliary. Moreover, to date only 6 natural products from the grayanane family were synthesized, out of the more than 160 compounds known to date. Thus, we anticipate that in the future, organic chemists will keep focusing on highly enantioselective, efficient and flexible synthetic strategies towards grayanane natural products.
